# Well-circumscribed type of intramuscular lipoma in the chest wall

**DOI:** 10.1186/1749-8090-8-181

**Published:** 2013-08-06

**Authors:** Jang-Hoon Lee, Hyung-Dong Do, Jung-Cheul Lee

**Affiliations:** 1 Department of Thoracic and Cardiovascular Surgery, College of Medicine, Yeungnam University, Daemyeong 5-dong, Nam-gu, Daegu, Korea

**Keywords:** Intramuscular lipoma, Thoracoscopic surgery

## Abstract

A tumor shadow was identified in the chest X-ray of a 40-year-old Korean man and he was referred to our hospital. The computed tomographic (CT) scan of his chest showed a 3-cm rounded pleural-based mass lesion with calcification, which was growing into the intercostal muscles. Thoracoscopic surgery was performed to resect the tumor. From the histological findings, the tumor was diagnosed as an intramuscular lipoma. The patient displayed no evidence of recurrence for more than 18 months. As well-circumscribed type of intramuscular lipoma is a rare tumor, we report this case with a literature review in this paper.

## Background

Lipoma is the most common form of soft tissue tumors. Intramuscular lipoma accounts for less than 1 percent of all the lipomas diagnosed [1]. This type of lipoma in the chest wall is rare and only a very few cases are known until now [2,3]. We herein report a case of a well-circumscribed type of intramuscular lipoma in the chest wall of a 40-year-old man.

## Case presentation

**Figure 1:**
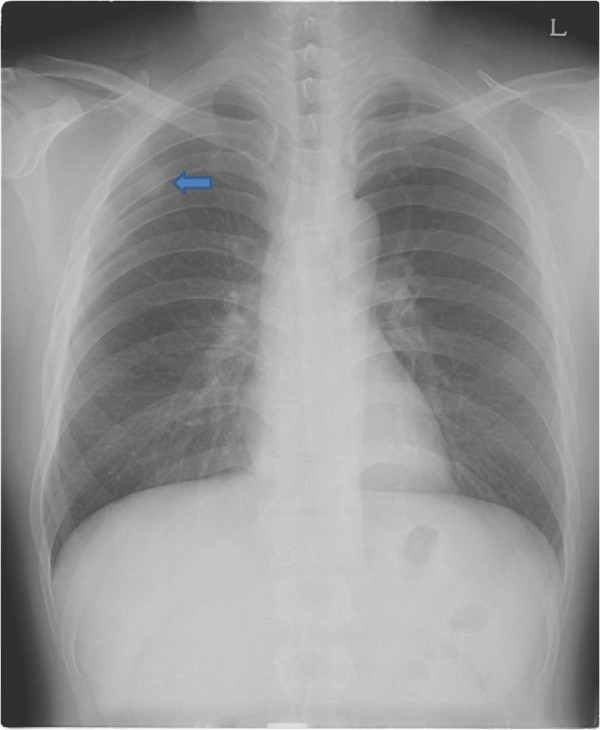
Preoperative chest radiograph (PA) view shows a round opacity on the right upper lung field (arrow).

**Figure 2:**
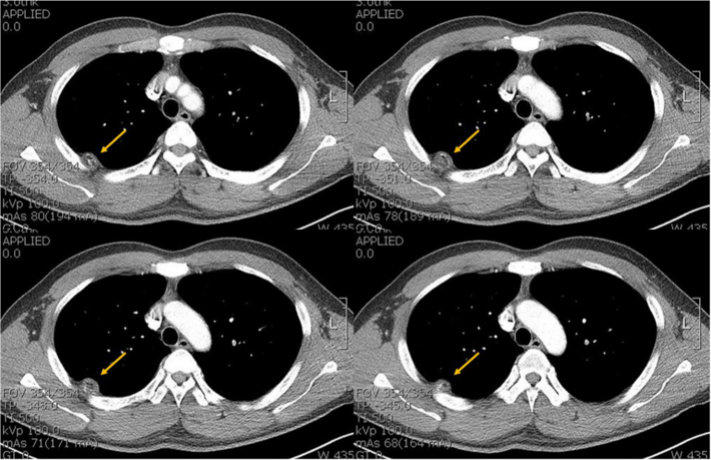
Contrast-enhanced computed tomography (CT) demonstrates the intrathoracic fatty mass with calcification that has obtuse margin projecting into the right hemithorax (arrow).

**Figure 3:**
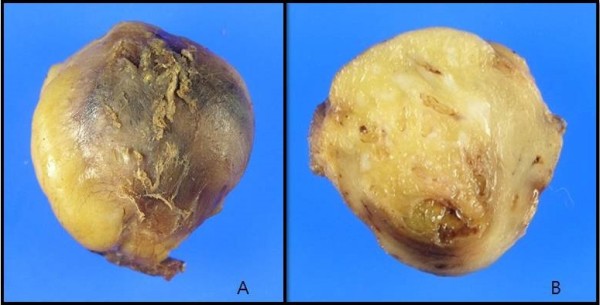
**Gross findings of the specimen. A**: The well-capsulated yellowish fatty mass measuring 3.8 X 2.5 X 2.0 cm and weighing 5.0 g was growing into the right fifth intercostal muscle. **B**: The cut surface of the lesion is well circumscribed, yellow soft, with focally gray-white areas.

**Figure 4:**
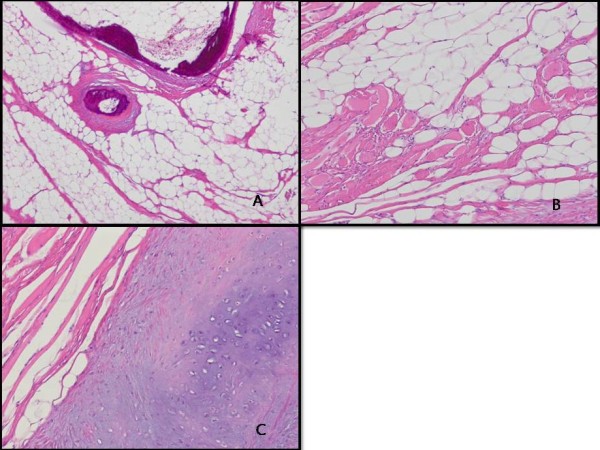
**Pathologic findings. A**: Mature adipocytes and focal ossification are seen (hematoxylin and eosin stain, x40). **B**: Entrapped skeletal muscle fibers are present (hematoxylin and eosin stain, x100). **C**: Chondroid metaplasia is noted (hematoxylin and eosin stain, x100).

A 40-year-old Korean man presented with a pulmonary nodule, which was detected by chest radiographs, and visited our hospital. He did not show any subjective symptoms. A 3-cm round mass lesion in the right upper lung field was seen in his chest X-ray (Figure 1), and the computed tomographic (CT) scan of his chest showed a 3.8 × 2.5 cm sized predominantly fatty mass with areas of calcification (Figure 2). The tumor was found to be lying in the right fifth intercostal space, which was well encapsulated but growing into intercostal muscles, and was resected completely by thoracoscopic surgery (Figure 3). Histopathological examination of the tumor confirmed it to be an intramuscular lipoma with chondroid metaplasia (Figure 4). Postoperative course of the patient was uneventful and he was discharged from the hospital on the fifth postoperative day. There was no evidence of recurrence for more than 18 months seen in the patient.

## Discussions

Lipoma, the most common form of soft tissue tumors, is composed of mature adipose tissue. Benign lipomatous tumors are divided into nine subtypes: lipoma, lipomatosis, lipomatosis of nerve, lipoblastoma/lipoblastomatosis, angiolipoma, myolipoma of soft tissue, chondroid lipoma, spindle-cell lipoma/pleomorphic lipoma, and hibernoma [4]. Intramuscular lipomas are found to occur preferentially in the lower extremity, with the trunk being the next most common location, followed by the shoulder girdle and upper extremity [5]. Intramuscular lipomas may be divided into well-circumscribed and infiltrative types. The infiltrative type represents 83% of the lipomas diagnosed and it typically invades the muscle fibers, eventually replacing them. In contrast, the well-circumscribed type lipoma presents with a distinct boundary and it is clearly distinguished from adjacent muscle cells [6]. As the intramuscular lipoma in our study was well capsulated and relatively easy resected, we categorized it to be the well-circumscribed type of intramuscular lipoma. Intramuscular lipomas are of primary importance because of their differential diagnosis with liposarcomas. They could be differentiated histologically from lipoblasts by their atypical nucleus, mucous degeneration, polymorphism, and mitosis [7]. Therefore, a detailed histological examination is essential to characterize intramuscular lipomas. Surgical excision is the best way to treat intramuscular lipoma. The recurrence rate for infiltrating lipomas has been reported to range between 3 and 62.5% [8,9]. This difference may be explained by inadequate resection of the tumor or misdiagnosing it as an intramuscular lipoma.

## Conclusions

The diagnosis of intramuscular lipoma is suggested when a painless, slow-growing, well-demarcated lesion occurs in the chest, although this type of lesion is rare. The liposarcoma that has a same histologic and clinical appearance must be excluded and surgical excision should be carefully performed. Patient follow-up is of critical importance due to its high recurrence rate.

## Consent

Written informed consent was obtained from the patient for publication of this case report and the accompanying images. A copy of the written consent is available for review by the Editor-in-Chief of this journal.

## Competing interests

The authors declare that they have no competing interests.

## Authors’ contributions

JHL, DHD, and JCL wrote the draft of the manuscript and obtained the written consent. JHL and DHD performed the literature review and participated in the manuscript writing and helped to the final writing of the paper and gave final approval of the manuscript. JHL performed the manuscript review and participated in the manuscript revision. All authors have read and approved the final manuscript.

## References

[B1] Myhre-Jensen O A consecutive 7-year series of 1331 benign soft tissue tumours. Clinicopathologic data. Comparison with sarcomas Acta Orthop Scand 1981 52 287 293 10.3109/17453678109050105 7282321

[B2] Carr CS Rawlins R O’Keefe PA Intramuscular lipoma of the intercostals muscle-the source of hour-glass transmural thoracic lipomas? Eur J Cardiothorac Surg 2001 19 361 10.1016/S1010-7940(01)00596-6 11251281

[B3] Takamori S Miwa K Hayashi A Shirouzu K Intramuscular lipoma in the chest wall Eur J Cardiothorac Surg 2004 26 1038 10.1016/j.ejcts.2004.07.042 15519201

[B4] Fletcher CD Unni KK Mertens F WHO classification of tumors. Pathology and genetics: tumors of soft tissue and bone 2002 Lyon, France: IARC Press 19 34

[B5] Laura WB Mark JK Jeffrey JP Mary IO Benign fatty tumors: classification, clinical course, imaging appearance, and treatment Skeletal Radiol 2006 35 719 733 10.1007/s00256-006-0189-y 16927086

[B6] Fletcher CD Martin-Bates E Intramuscular and intermuscular lipoma: neglected diagnosis Histopathology 1998 12 275 287 3366443 10.1111/j.1365-2559.1988.tb01942.x

[B7] Sohn WI Kim JH Jung SN Kwon H Cho KJ Intramuscular lipoma of the sternocleidomastoid muscle J craniofac surg 2010 21 1976 1978 10.1097/SCS.0b013e3181f502cd 21119474

[B8] Jonathan JD John DW Intramuscular lipoma of the superior oblique muscle Orbit 2006 25 227 233 10.1080/01676830600575519 16987771

[B9] Akbulut M Aksoy A Bir F Intramuscular lipoma of the tongue: a case report and review of the literature Aegean Pathology Journal 2005 2 146 149

